# Utility and predictive value of the CRAFITY score in advanced hepatocellular carcinoma treated with transarterial chemoembolization plus tyrosine kinase inhibitors and PD-1 inhibitor

**DOI:** 10.1186/s12885-024-11936-0

**Published:** 2024-02-16

**Authors:** Lijie Zhang, Tao Sun, Bo Sun, Kailu Zhang, Yuting Zheng, Na Li, Lei Chen, Chuansheng Zheng, Bin Liang, Heshui Shi

**Affiliations:** 1 Department of Radiology, Union Hospital, Tongji Medical College, Huazhong University of Science and Technology, 1277 Jiefang Road, 430022 Wuhan, China; 2 Hubei Province Key Laboratory of Molecular Imaging, 430022 Wuhan, China

**Keywords:** Hepatocellular carcinoma, Transarterial chemoembolization, Tyrosine kinase inhibitors, PD-1 inhibitor

## Abstract

**Background:**

The prognostic significance of the CRAFITY score (**CR**P and **AF**P in **I**mmuno**T**herap**Y**) has been demonstrated in hepatocellular carcinoma (HCC) patients receiving immunotherapy. The purpose of this study was to investigate the utility and the predictive value of CRAFITY score in HCC after transarterial chemoembolization (TACE) in combination with tyrosine kinase inhibitors (TKIs) and immunotherapy.

**Materials and methods:**

Data from patients with advanced HCC treated with TACE plus TKIs and PD-1 inhibitor from January 2019 to June 2022 were collected and analyzed retrospectively. Patients with AFP ≥ 100 ng/mL and those with CRP ≥ 1 mg/dL were assigned a CRAFITY score of 1 point. Patients were divided into three groups according to their CRAFITY score (CRAFITY-low, 0 points; CRAFITY-intermediate, 1 point; and CRAFITY-high, 2 points). The differences in overall survival (OS), progression-free survival (PFS) and adverse events (AEs) were compared among the three groups. Tumor response was evaluated at 3, 6 and 12 months after the first combination treatment. Risk factors for OS and PFS were assessed.

**Results:**

A total of 70 patients were included. The patients were assigned CRAFITY scores of 0 points (CRAFITY-low, *n* = 25 [35.71%]), 1 point (CRAFITY-intermediate, *n* = 29 [41.42%]), and 2 points (CRAFITY-high, *n* = 16 [22.81%]). Multivariate analysis showed that lower CRAFITY score was an independent factor for the improved OS (*P* =.045) and PFS (*P* <.001). TACE session was also associated with the OS (*P* =.048) in the multivariate analysis. The CRAFITY-low cohort achieved a higher objective response rate (ORR) at the 3-month evaluation of tumor response. However, there was no significant difference in ORR and disease control rate (DCR) observed at the 6-month follow-up. DCR showed a statistically significant difference among three groups during the 12-month follow-up period. The percentage of patients with protein urea was highest in the CRAFITY-high group. No significance differences were observed in grade ≥ 3 AEs in three groups.

**Conclusion:**

The CRAFITY score is simple and could be useful for predicting treatment outcomes, tumor response and AEs of the HCC patients receiving TACE plus TKIs and PD-1 inhibitor therapy.

## Introduction

Liver cancer is one of the most common malignancies worldwide with approximately 70% of new cases diagnosed in Asia [1]. Hepatocellular carcinoma (HCC) represents about 80% of primary liver cancer and develops in the background of chronic liver diseases [2]. Surgery, ablation and liver transplantation are the definitive treatments for HCC, however, are only suitable for about 20% of patients [3, 4]. Transarterial chemoembolization (TACE) has been recommended as an effective palliative therapy for patients with unresectable HCC and exhibited encouraging survival outcomes [5].

Systemic therapies, such as tyrosine kinase inhibitors (TKIs), and immunotherapy, including immune checkpoint inhibitors (ICIs), have revolutionized the treatment landscape of conventional therapies for HCC [2]. TKIs can inhibit tumor angiogenesis and vascular abnormalities, leading to cancer cell death and tumor shrinkage. On the other hand, ICIs can block immunosuppressive pathways, allowing the immune system to effectively eliminate malignant tumors [5]. Additionally, emerging evidence suggests that combining TACE with PD-1/PD-L1 inhibitors and TKIs has shined a light to the HCC patients [6]. While most research focuses on assessing the effectiveness of this combination therapy, the specific patient population that benefits from it remains uncertain due to tumor heterogeneity. Furthermore, few clinical trials have identified predictors of successful outcomes for combination therapy.

Scheiner et al. [7] reported that a combination of alpha-fetoprotein (AFP) and C-reactive protein (CRP) has the potential to predict HCC response to immunotherapy, termed the CRAFITY score (**CR**P and **AF**P in **I**mmuno**T**herap**Y**). In this clinical score, AFP > 100 ng/mL and CRP > 1 mg/mL was assigned a point of 1, respectively, so the cohort was divided into three groups with a score of 0 (AFP < 100 ng/mL, CRP < 1 mg/mL), 1 (AFP > 100 ng/mL or CRP > 1 mg/mL) or 2 (AFP > 100 ng/mL and CRP > 1 mg/mL). In the training cohort (*n* = 190), higher CRAFITY scores were significantly associated with the shorter OS and non-response in radiology, which was consistent with the results in the validation set (*n* = 102). The predictive value of the CRAFITY score for TKIs plus ICIs combinations was also examined in another HCC cohorts and yield excellent prognostic efficacy [8].

Therefore, this research aimed to investigate the utility and predictive value of the CRAFITY score in HCC patients receiving combined of TACE plus ICIs and TKIs therapy. We performed a single center retrospective study to broaden the use of the CRAFITY score.

## Methods

### Patients

A retrospective analysis was conducted of consecutive HCC patients who received TACE plus ICIs and TKIs therapy in our institution from January 2019 to June 2022.

The eligibility criteria for the present study were as follows: (1) patients aged > 18 years; (2) the confirmed diagnosis of HCC by pathological or clinical diagnosis according to EASL criteria [5], accompanied by macrovascular invasion and/or extrahepatic metastasis; (3) patients received the combination of TACE plus ICIs and TKIs as the treatment strategy; (4) full record of the serum levels of AFP and CRP at baseline; (5) liver function Child-Pugh Class A or B without ascites and (6) Eastern Cooperative Oncology Group (ECOG) performance status of 0–2.

The exclusion criteria were: (1) history of organ transplantation; (2) previously received other treatments, such as TACE, hepatic arterial infusion chemotherapy (HAIC), radiotherapy or systemic therapy; (3) recurrence HCC after curative resection or ablation; (4) technically unsuccessful TACE procedure; and (5) irregular follow-up or lost to follow-up within 3 months after TACE operation. Patients were categorized into three groups according to their CRAFITY score (CRAFITY-low, 0 points; CRAFITY-intermediate, 1 point; and CRAFITY-high, 2 points).

### TACE treatment

Conventional TACE (C-TACE) or TACE with CalliSpheres® microspheres (CSM-TACE) was performed in three cohorts. A 5 F visceral catheter was introduced to catheterize the celiac trunk and the superior mesenteric artery after vascular access through the common femoral artery. Selective arteriography was performed to detect potential hypervascular tumors. The potential extrahepatic collateral vessels were interrogated to exclude malignant parasitization of blood flow if necessary. A 2.7 F coaxial microcatheter system (Progreat, Terumo, Tokyo, Japan) was advanced into the tumor-feeding arteries.

For C-TACE, the emulsion of 5–20 mL lipiodol (Lipiodol Ultrfluido, Guerbet, Paris, France) mixed with 20–60 mg epirubicin (Hisun Pfizer Pharmaceutical Co., Ltd., Zhejiang, China) was used as chemoembolization agents, followed by embolization with embolic materials.

For CSM-TACE, the CalliSpheres® Beads (Jiangsu Hengrui Medicine Co. Ltd., Jiangsu, China) with 100–300 μm or 300–500 μm in diameter, which was loaded with epirubicin (50 or 80 mg) were used as the drug carrier and embolization agent.

Technical success was defined as successful advancement of a catheter into tumor-feeding arteries and transarterial therapy administration according to the investigator-designated plan and would be assessed for each TACE procedure. For bilobar or huge lesions, at least two TACE sessions 4–6 weeks apart were required to achieve complete treatment.

### Systemic treatment

Written informed consent was obtained from the parents of each patient prior to systemic treatment. Sorafenib (Nexavar®, Bayer AG, Leverkusen, Germany) was administered at a dose of 400 mg orally, twice per day. The dose of Lenvatinib (Lenvima; Eisai Co., Ltd., Tokyo, Japan) was 8 mg orally, once per day (body weight < 60 kg) or 12 mg orally, once per day (body weight ≥ 60 kg). Apatinib (Jiangsu Hengrui Medicine Co., Ltd., Jiangsu, China) was initiated at an oral dose of 500 mg/day. The programmed death 1 (PD-1) inhibitor sintilimab (Innovent Biologics Co., Ltd., Jiangsu, China) or camrelizumab (Jiangsu Hengrui Medicine Co., Ltd., Jiangsu, China) was injected intravenously at 200 mg once every 3 weeks.

The systemic therapy was initiated within 7 days after the first TACE operation and discontinued for 2–3 days before and after TACE. The interruption, dose reduction and discontinuation of drug administration depended on the development of disease progression (PD) or the presence of unacceptable adverse events (AEs).

### Follow-up

Selected patients were followed up regularly every 4–6 weeks. During follow-up, a detailed out-of-hospital history, laboratory tests, and CT or MRI imaging were requested. The systemic therapy was discontinued in the patients with severe toxicity, PD or the change of treatment strategy. The subsequent treatment, such as the second-line drug or in combination with other treatments, was determined with the discussion of the multidisciplinary team.

### Efficacy and safety

Overall survival (OS) was the primary endpoint of this study, defined as the time from initiation of the combination treatment to death. The secondary endpoint was progression-free survival (PFS), defined as the time from the treatment initiation to either radiological progression or death. Censored patients were defined as the patients who remained alive at the end of follow-up or who were lost to follow-up within 3 months after the combination treatment. Tumor response was evaluated on contrast-enhanced CT or MRI obtained at 3, 6 and 12 months after TACE. All images were assessed for tumor characteristics and tumor response by consensus of two radiologists (Bin Liang and Heshui Shi) in a blinded fashion. Tumor response, including complete response (CR), partial response (PR), stable disease (SD) and progressive disease (PD), was measured according to the modified Response Evaluation Criteria in Solid Tumors (mRECIST) scheme [9]. For the patients with PD, the evaluation would be conducted again to confirm the occurrence of immune-related confirmed progressed disease (iCPD) by immune-related response criteria in solid tumors (iRECIST) [10]. Objective response rate (ORR) was defined as the sum of CR and PR. Disease control rate (DCR) was defined as the sum of CR, PR and SD.

AEs related to the therapy were assessed and recorded based on National Cancer Institute Common Terminology Criteria for Adverse Events version 5.0. The postembolization syndrome usually presented with fever, nausea or vomiting and pain. The syndrome by itself is not considered an adverse event, but rather an expected outcome of embolization. A severe adverse event is any event that results in additional therapy, including an increased level of care, hospital stay beyond observation status (including readmission after initial discharge), permanent adverse sequelae including substantial morbidity and disability, and death.

### Statistical analysis

Categorical data were expressed as number of patients (percentage). Continuous data were expressed as mean ± standard deviation and median (range) for normally and nonnormally distributed variables, respectively. The Wilcoxon Signed Rank Test, Kruskal-Wallis test, Pearson Chi-square test and Fisher Exact test were used to compare variables, as appropriate. The OS and PFS were evaluated using the Kaplan–Meier method, and 95% confidence interval (CI) were provided for proportions. Variables associated (*P* ≤.4) with survival at univariate analysis were entered in the Cox multivariate stepwise regression model to identify the independent prognostic factors. The adjusted relative risk (hazard ratio, HR) and 95% CI were calculated for each independent predictive factor. All statistical analyses were performed by using software (SPSS, version 24.0; SPSS Inc., Chicago, Illinois, USA). A two-tailed *P* value less than 0.05 was considered to indicate statistically significant differences. Graphs in this study were created using GraphPad Prism 8.0 (GraphPad Software Inc., San Diego, CA, USA).

To identify factors that might predict OS and PFS, the following variables were analyzed: CRAFITY score, age, gender, pathogeny, comorbidity, Eastern Cooperative Oncology Group (ECOG) score, Child-Pugh class, alanine aminotransferase (ALT), aspartate aminotransferase (AST), platelet/lymphocyte ratio (PLR), neutrophil/lymphocyte ratio (NLR), types of TKIs, TACE sessions and tumor characteristics, including Barcelona Clinic Liver Cancer (BCLC) stage, tumor number and tumor size.

## Results

### Patient characteristics

A total of 70 HCC patients were included during the study period. The average age of all patients was 54.0 (28.0–72.0) years old, and 58 cases (82.86%) were male. The patients (*n* = 54 [77.14%]) were usually infected by hepatitis-B virus in this study. There were 17 patients treated with sorafanib, 26 patients receiving apatinib and 27 patients prescribed oral lenvatinib. Regarding ICIs, 60 patients received camrelizumab therapy and 10 patients were treated with sintilimab injection. There was no statistical difference in the treatment regimens among the three groups. There were 24 (34.29%) patients with serum CRP ≥ 1.0 mg/dL, and 36 (51.43%) patients with a serum AFP ≥ 100 ng/mL. Accordingly, these patients were assigned CRAFITY scores of 0 (CRAFITY-low, *n* = 25 [35.71%]), 1 (CRAFITY-intermediate, *n* = 29 [41.43%]), and 2 points (CRAFITY-high, *n* = 16 [22.86%]). There were no significant differences in BCLC stage, tumor number, and tumor size among the three cohorts. The demographic and clinical characteristics of the recruited patients are summarized in Table 1.

**Table 1:** Demographic and clinical characteristics of patients between the different groups

Variables	All patients (*n* = 70) ^*^	CRAFITY-low (*n* = 25) ^*^	CRAFITY-intermediate (*n* = 29) ^*^	CRAFITY-high (*n* = 16) ^*^	*P* value
Age (yrs)	53.84 ± 10.37	52.00 ± 11.88	53.69 ± 10.35	57.00 ± 7.23	0.557
Gender					0.305
Male	58 (82.86%)	23 (92.00%)	22 (75.86%)	13 (81.25%)	
Female	12 (17.14%)	2 (8.00%)	7 (24.14%)	3 (18.75%)	
Pathogeny					0.712
HBV-related	54 (77.14%)	20 (80.00%)	23 (79.31%)	11 (68.75%)	
Others	16 (22.86%)	5 (20.00%)	6 (20.69%)	5 (31.25%)	
ECOG Score					0.647
0	47 (67.14%)	15 (60.00%)	21 (72.41%)	11 (68.75%)	
1	23 (32.86%)	10 (40.00%)	8 (27.59%)	5 (31.25%)	
Child-Pugh class					0.184
A	56 (80.00%)	20 (80.00%)	26 (89.66%)	11 (68.75%)	
B	14 (20.00%)	5 (20.00%)	3 (10.34%)	5 (31.25%)	
ALT (u/L)	38.11 ± 23.83	32.44 ± 19.49	42.69 ± 21.90	38.69 ± 31.85	0.203
AST (u/L)	57.99 ± 62.18	44.28 ± 22.27	70.66 ± 86.57	56.44 ± 48.72	0.379
AFP (ng/mL)					< 0.001
< 100	34 (48.8%)	25 (100.00%)	9 (31.03%)	0 (0.00%)	
≥ 100	36 (51.2%)	0 (0.00%)	20 (68.97%)	16 (100.00%)	
CRP (mg/dL)	0.59 ± 0.51	0.18 ± 0.12	0.57 ± 0.45	1.26 ± 0.19	< 0.001
PLR	149.36 ± 97.96	113.27 ± 58.64	167.05 ± 110.44	173.66 ± 111.67	0.092
NLR	3.33 ± 2.82	2.61 ± 1.70	3.97 ± 3.76	3.30 ± 2.01	0.260
Types of TKIs					0.461
Sorafanib	17 (24.29%)	7 (28.00%)	4 (13.79%)	6 (37.50%)	
Apatinib	26 (37.14%)	8 (32.00%)	13 (44.83%)	5 (31.25%)	
Lenvatinib	27 (38.57%)	10 (40.00%)	12 (41.38%)	5 (31.25%)	
Types of ICIs					0.829
camrelizumab	60 (85.71%)	22 (88.00%)	25 (86.21%)	13 (81.25%)	
sintilimab	10 (14.29%)	3 (12.00%)	4 (13.79%)	3 (18.75%)	
TACE sessions	4.11 ± 2.88	4.80 ± 2.91	3.48 ± 1.66	4.19 ± 4.23	0.221
BCLC stage					0.109
B	28 (40.00%)	14 (56.00%)	10 (34.48%)	4 (25.00%)	
C	42 (60.00%)	11 (44.00%)	19 (65.52%)	12 (75.00%)	
Tumor number					0.150
1	24 (34.28%)	11 (44.00%)	6 (20.69%)	7 (43.75%)	
≥ 2	46 (65.72%)	14 (56.00%)	23 (79.31%)	9 (56.25%)	
Tumor size (cm)					0.098
≤ 5	17 (24.29%)	5 (20.00%)	7 (24.14%)	5 (31.25%)	
5 ~ 10	33 (47.14%)	15 (60.00%)	15 (51.72%)	3 (18.75%)	
≥ 10	20 (28.57%)	5 (20.00%)	7 (24.14%)	8 (50.00%)	

HBV, Hepatitis B virus infection; ECOG, Eastern Cooperative Oncology Group; ALT, alanine aminotransferase; AST, aspartate aminotransferase; AFP, alpha-fetoprotein; CRP, C-reactive protein; PLR, platelet/lymphocyte ratio; NLR, neutrophil/lymphocyte ratio; TKIs, tyrosine kinase inhibitors; TACE, transarterial chemoembolization; BCLC Barcelona Clinic Liver Cancer

^*^ Except where indicated, data are numbers of patients, with percentages in parentheses, or means ± standard deviations

### Treatment process

Technically successful TACE treatments were performed in all patients. The patients underwent a total of 283 TACE procedures, including 254 C-TACE procedures and 29 CSM-TACE procedures. A median of 3 (range 1–12) TACE treatments were received per patient. Specifically, the patients in the CRAFITY-low group underwent 120 TACE procedures with a median number of 4 (range, 1–11), including 114 C-TACE procedures and 7 CSM-TACE procedures. In the CRAFITY-intermediate group, the 29 patients underwent a total of 101 TACE treatments with a median of 3 (range 1–7), including 86 C-TACE operations and 15 CSM-TACE sessions. As for CRAFITY-high group, 16 patients received 52 C-TACE sessions, while 6 of these accepted 8 CSM-TACE procedures. Totally, all the patients in this group underwent 60 TACE procedures. The median number of TACE treatment was 2 (range, 2–12).

In terms of the systemic therapy administration, grade 3 adverse reactions occurred in 8 patients, and the dose of oral TKI was reduced to half of the respective standard dose. Among them, 4 cases were in the CRAFITY-low group, 2 of them received sorafenib therapy and the others were treated with apatinib. Another 3 patients were in the CRAFITY-intermediate group, 2 of them received sorafenib therapy and 1 patient accepted apatinib therapy. Only 1 patient in the CRAFITY-high group received dose reduction of sorafenib and the antihypertensive drugs. The administration of lenvatinib was interrupted in 1 patient from CRAFITY-high group due to the severe proteinuria, and the second-line therapy was initiated. There were no changes to the protocol for the administration of ICIs injections.

### Follow up and tumor response

The median follow-up period was 14.0 months (range, 3–29 months). At 3-month evaluation, in the CRAFITY-low group, the CR was achieved in 5 patients, a PR in 12 patients, SD in 6 patients, and PD in 2 patients, while, that was 1 patient, 13 patients,14 patients and 1 patient in CRAFITY-intermediate group, respectively. There were 0, 4, 9 and 3 patients in the CRAFITY-high group had CR, PR, SD and PD, respectively. For patients with PD, 2 patients were confirmed as iCPD (1 patient in CRAFITY-low group and 1 patient in CRAFITY-high group). A higher ORR was achieved in the CRAFITY-low group when compared with the other two groups (*P* =.026), while no significance difference in the DCR. Tumor responses of the three groups were shown in Table 2.

**Table 2:** Tumor response at 3 months after the combination therapy

Response	Patients, No. (%)			*P* value
	CRAFITY-low (*n* = 25) ^*^	CRAFITY-intermediate (*n* = 29) ^*^	CRAFITY-high (*n* = 16) ^*^	
CR	5 (40.00%)	1 (3.45%)	0 (00.00%)	-
PR	12 (48.00%)	13 (44.83%)	4 (25.00%)	-
SD	6 (24.00%)	14 (48.28%)	9 (56.25%)	-
PD	2 (8.00%)	1 (3.45%)	3 (18.75%)	-
ORR	17 (68.00%)	14 (48.28%)	4 (25.00%)	0.026
DCR	23 (92.00%)	28 (96.55%)	13 (81.25%)	0.212

^*^ Data are presented as n (%). CR, complete response; PR, partial response; SD, stable disease; PD, progressive disease; ORR, objective response rate

At the 6-month evaluation, four patients died in the CRAFITY-high group. There were no significant differences observed in both the ORR and DCR (Table 3). At the 12-month evaluation of tumor response, there were 4, 11, and 13 patient deaths in the three groups, respectively. The DCR showed statistical significance, while the ORR did not (Table 4).

**Table 3:** Tumor response at 6 months after the combination therapy

Response	Patients, No. (%)			*P* value
	CRAFITY-low (*n* = 25) ^*^	CRAFITY-intermediate (*n* = 29) ^*^	CRAFITY-high (*n* = 12) ^*^	
CR	7 (28.00%)	5 (17.24%)	1 (8.33%)	-
PR	13 (52.00%)	16 (55.17%)	5 (41.67%)	-
SD	4 (16.00%)	6 (20.69%)	4 (33.33%)	-
PD	1 (4.00%)	2 (6.90%)	2 (16.67%)	-
ORR	20 (80.00%)	21 (72.41%)	6 (50.00%)	0.165
DCR	24 (96.00%)	27 (93.10%)	10 (83.33%)	0.388

^*^ Data are presented as n (%). CR, complete response; PR, partial response; SD, stable disease; PD, progressive disease; ORR, objective response rate

**Table 4:** Tumor response at 12 months after the combination therapy

Response	Patients, No. (%)			*P* value
	CRAFITY-low (*n* = 21) ^*^	CRAFITY-intermediate (*n* = 18) ^*^	CRAFITY-high (*n* = 3) ^*^	
CR	6 (28.57%)	8 (44.44%)	0 (0.00%)	-
PR	10 (47.62%)	6 (33.33%)	2 (66.67%)	-
SD	4 (19.05%)	3 (16.67%)	1 (33.33%)	-
PD	1 (4.76%)	1 (5.56%)	0 (0.00%)	-
ORR	16 (76.19%)	14 (77.77%)	2 (66.67%)	0.916
DCR	20 (95.24%)	17 (94.44%)	3 (100.00%)	0.005

^*^ Data are presented as n (%). CR, complete response; PR, partial response; SD, stable disease; PD, progressive disease; ORR, objective response rate

### Risk factors analysis

In the univariate analysis, CRAFITY score (*P* =.156), Age (*P* =.207), Child-Pugh class (*P* =.280), ALT (*P* =.336), AST (*P* =.290), PLR (*P* =.332), Types of TKIs (*P* =.188), TACE sessions (*P* =.049), tumor number (*P* =.302) and tumor size (*P* =.230) were considered as the potential risk factors of OS. For PFS, CRAFITY score (*P* <.001), ALT (*P* =.325), AST (*P* =.072), PLR (*P* =.013), NLR (*P* =.244), BCLC stage (*P* =.138) and tumor size (*P* =.105) were significant factors. In the multivariate analysis, however, only CRAFITY score (*P* =.045) and TACE sessions (*P* =.048) showed significance for OS after adjustment for other variables. For PFS, only CRAFITY score (*P* <.001) was the independent factors (Tables 5 and 6).

**Table 5:** Results of univariate and multivariate analysis of factors associated with OS

Parameters	Univeriate analysis ^#^				Multiveriate analysis		
	HR	(95%CI)	*P* Value		HR	(95%CI)	*P* Value
CRAFITY score	1.736	0.810–3.720	0.156		3.429	1.026–11.455	0.045
Age	1.024	0.987–1.062	0.207		1.026	0.976–1.079	0.313
Gender	0.569	0.126–2.570	0.464				
Pathogeny	1.114	0.364–3.411	0.851				
ECOG score	1.281	0.394–4.165	0.680				
Child-Pugh class	0.038	0.000-14.200	0.280		0.000	0.000-1.475	0.968
ALT	1.011	0.989–1.033	0.336		1.005	0.974–1.038	0.736
AST	1.008	0.994–1.022	0.290		1.011	0.984–1.038	0.438
PLR	1.003	0.997–1.009	0.332		0.996	0.987–1.005	0.405
NLR	0.972	0.788–1.198	0.788				
Types of TKIs	0.678	0.381–1.208	0.188		0.524	0.218–1.258	0.148
TACE sessions	1.161	1.001–1.347	0.049		1.244	1.001–1.546	0.048
BCLC stage	1.347	0.539–3.365	0.524				
Tumor number	1.292	0.794–2.102	0.302		0.867	0.469–1.602	0.649
Tumor size	1.559	0.755–3.223	0.230		2.844	0.971–8.328	0.056

^#^ variables with *P* value ≤ 0.4 in the univariate analysis were further included in the multivariate Cox proportional hazards regression model analysis. OS, overall survival; ECOG, Eastern Cooperative Oncology Group ALT, alanine aminotransferase; AST, aspartate aminotransferase; PLR, platelet/lymphocyte ratio; NLR, neutrophil/lymphocyte ratio; TKIs, tyrosine kinase inhibitors; TACE, transarterial chemoembolization; BCLC Barcelona Clinic Liver Cancer

**Table 6:** Results of univariate and multivariate analysis of factors associated with PFS

Parameters	Univeriate analysis ^#^				Multiveriate analysis		
	HR	(95%CI)	*P* Value		HR	(95%CI)	*P* Value
CRAFITY score	2.412	1.618–3.595	< 0.001		2.279	1.454–3.571	< 0.001
Age	1.004	0.981–1.027	0.739				
Gender	1.076	0.525–2.203	0.841				
Pathogeny	0.895	0.487–1.645	0.720				
ECOG score	1.046	0.594–1.840	0.876				
Child-Pugh class	0.963	0.451–2.056	0.922				
ALT	1.005	0.995–1.016	0.325		0.996	0.982–1.010	0.553
AST	1.003	1.000-1.007	0.072		1.003	0.997–1.009	0.342
PLR	1.004	1.001–1.006	0.013		1.000	0.995–1.004	0.985-
NLR	1.057	0.963–1.161	0.244		1.022	0.900–1.160	0.739
Types of TKIs	0.996	0.720–1.377	0.980				
TACE sessions	1.031	0.928–1.146	0.564				
BCLC stage	1.529	0.872–2.680	0.138		1.017	0.542–1.907	0.959
Tumor number	1.085	0.820–1.437	0.568				
Tumor size	1.433	0.928–2.213	0.105		1.287	0.777–2.133	0.328

^#^ variables with *P* value ≤ 0.4 in the univariate analysis were further included in the multivariate Cox proportional hazards regression model analysis. PFS, progression-free survival; ECOG, Eastern Cooperative Oncology Group ALT, alanine aminotransferase; AST, aspartate aminotransferase; PLR, platelet/lymphocyte ratio; NLR, neutrophil/lymphocyte ratio; TKIs, tyrosine kinase inhibitors; TACE, transarterial chemoembolization; BCLC Barcelona Clinic Liver Cancer

**Fig. 1:**
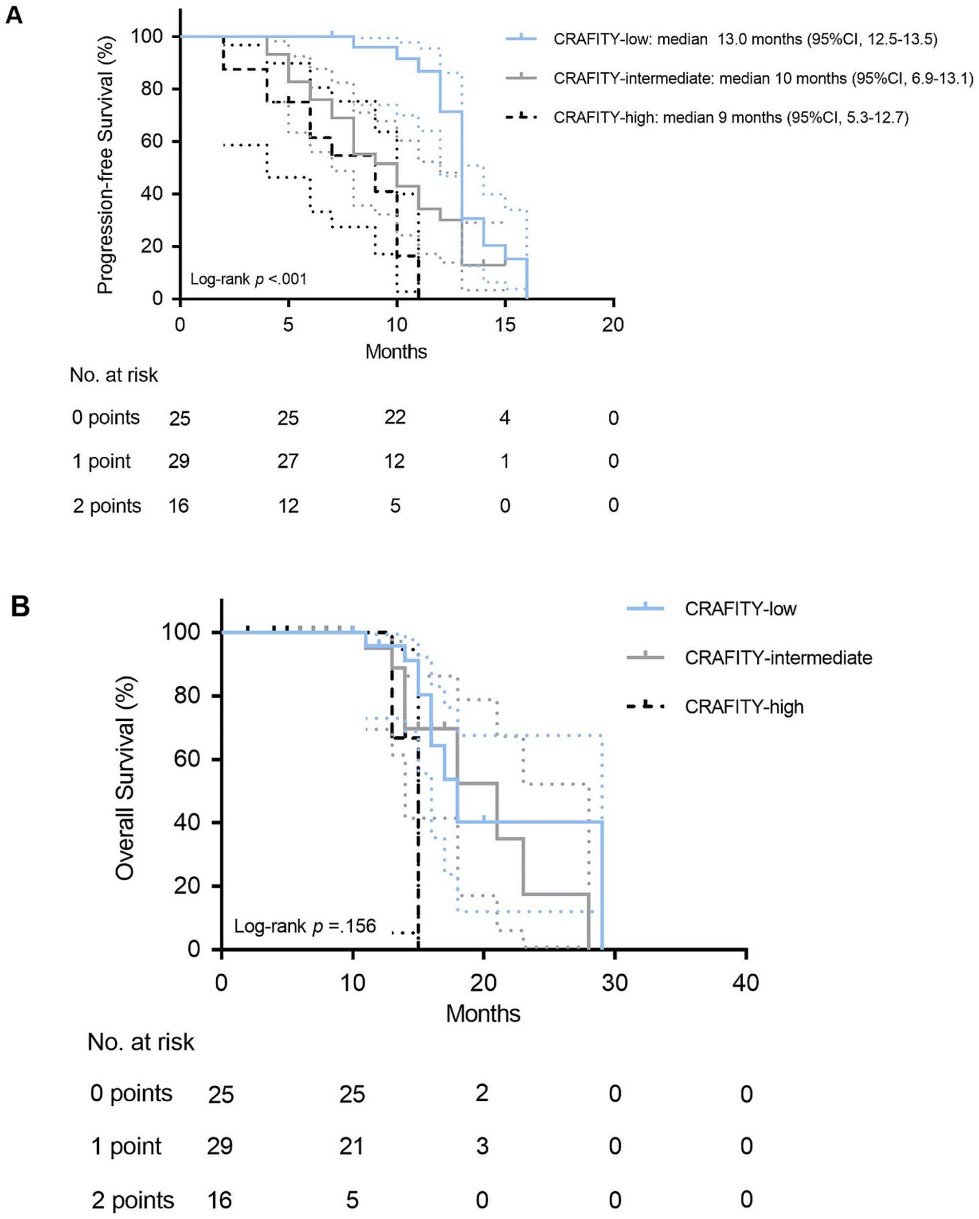
Kaplan-Meier analysis of PFS and OS by the CRAFITY score. **A**, Kaplan-Meier curves of PFS divided into three groups according to the CRAFITY score. **B**, Kaplan-Meier curves pf the OS according to the CRAFITY score. PFS, progression-free survival; OS, overall survival

The Kaplan-Meier curves of PFS according to CRAFITY score was divided into three groups. The median survival time of patients in CRAFITY-low group was 13.0 months (95% CI 12.5–13.5) and 10.0 months (95% CI 6.9–13.1) for patients in CRAFITY-intermediate group. Patients in CRAFITY-high cohort had a median survival of 9.0 months (95% CI 5.3–12.7). There was a significant difference between them (Fig. 1A). Figure 1B showed the Kaplan-Meier estimates of OS for the patients in three groups though the statistical significance was not indicated.

### Safety

The summary of AEs according to the CRAFITY score was shown in Table 7. In total, the treatment-related AEs were observed in 62 of the 70 patients (88.57%) and no permanent adverse sequelae including substantial morbidity and disability, and death occurred during the hospitalization and follow-up. The severity and frequency of AEs were similar between the three groups (any grade, *P* =.790; grade ≥ 3, *P* =.824). Postembolization syndrome, including abdominal pain (*P* =.714), fever (*P* =.906) nausea or vomiting (*P* =.878) and transaminitis (*P* =.829) were the most common TACE-related events and all the symptoms relieved with symptomatic treatment. For the systemic therapy-related AEs, significant differences were observed in any grade of protein urea (*P* =.012). The rate of protein urea was lowest in patients with a CRAFITY score of 0, followed by patients with CRAFITY scores of 1 and 2. Only 1 patient in the CRAFITY-high group had severe proteinuria. There were 4, 3 and 2 patients in the three groups developed severe secondary hypertension, respectively. No significance differences were observed in grade ≥ 3 AEs.

**Table 7:** Treatment-related AEs

Items		CRAFITY-low (*n* = 25)	CRAFITY-intermediate (*n* = 29)	CRAFITY-high (*n* = 16)	*P* Value
Total	Any	23 (92.00%)	25 (86.20%)	14 (87.50%)	0.790
	Grade ≥ 3	4 (16.00%)	3 (10.34%)	2 (12.50%)	0.824
TACE attributed					
Abdominal pain	Any	20 (80.00%)	22 (75.86%)	11 (68.75%)	0.714
	Grade ≥ 3	0 (00.00%)	0 (00.00%)	0 (00.00%)	-
Fever	Any	21 (84.00%)	23 (79.31%)	13 (81.25%)	0.906
	Grade ≥ 3	0 (00.00%)	0 (00.00%)	0 (00.00%)	-
Nausea/vomiting	Any	17 (68.00%)	21 (72.41%)	12 (75.00%)	0.878
	Grade ≥ 3	0 (00.00%)	0 (00.00%)	0 (00.00%)	-
Transaminitis	Any	22 (88.00%)	25 (86.21%)	13 (81.25%)	0.829
	Grade ≥ 3	0 (00.00%)	0 (00.00%)	0 (00.00%)	-
Systemic therapy attributed					
Fatigue	Any	6 (24.00%)	6 (20.69%)	8 (50.00%)	0.093
	Grade ≥ 3	0 (00.00%)	0 (00.00%)	0 (00.00%)	-
Secondary Hypertension	Any	10 (40.00%)	8 (27.58%)	5 (31.25%)	0.618
	Grade ≥ 3	4 (16.00%)	3 (10.34%)	2 (12.50%)	0.824
Hand-foot syndrome	Any	7 (28.00%)	5 (17.24%)	5 (31.25%)	0.495
	Grade ≥ 3	0 (00.00%)	0 (00.00%)	0 (00.00%)	-
Diarrhea/colitis	Any	2 (8.00%)	1(3.44%)	0 (00.00%)	0.447
	Grade ≥ 3	0 (00.00%)	0 (00.00%)	0 (00.00%)	-
Protein urea	Any	4 (16.00%)	4 (13.79%)	8 (50.00%)	0.012
	Grade ≥ 3	0 (00.00%)	0 (00.00%)	1 (6.25%)	0.180
Hypothyroidism	Any	1 (4.00%)	1 (3.45%)	0 (00.00%)	0.731
	Grade ≥ 3	0 (00.00%)	0 (00.00%)	0 (00.00%)	-
Constipation	Any	3 (12.00%)	5 (6.89%)	3 (18.75%)	0.809
	Grade ≥ 3	0 (00.00%)	0 (00.00%)	0 (00.00%)	-
Decreased appetite	Any	8 (32.00%)	11 (37.93%)	9 (75.00%)	0.289
	Grade ≥ 3	0 (00.00%)	0 (00.00%)	0 (00.00%)	-
Hypothyroidism	Any	0 (00.00%)	1 (3.45%)	0 (00.00%)	0.488
	Grade ≥ 3	0 (00.00%)	0 (00.00%)	0 (00.00%)	-
Rash	Any	1 (4.00%)	2 (6.89%)	1 (6.25%)	0.895
	Grade ≥ 3	0 (00.00%)	0 (00.00%)	0 (00.00%)	-
Gingival bleeding	Any	2 (8.00%)	2 (6.89%)	1 (6.25%)	0.975
	Grade ≥ 3	0 (00.00%)	0 (00.00%)	0 (00.00%)	-
Insomnia	Any	1 (4.00%)	2 (6.89%)	1 (6.25%)	0.895
	Grade ≥ 3	0 (00.00%)	0 (00.00%)	0 (00.00%)	-
Pneumonitis	Any	0 (00.00%)	1 (3.45%)	1 (6.25%)	0.488
	Grade ≥ 3	0 (00.00%)	0 (00.00%)	0 (00.00%)	-

AEs, adverse events; TACE, transarterial chemoembolization

## Discussion

To date, no suitable predictive molecular biomarkers have been demonstrated to predict the prognosis of HCC patients receiving TACE plus TKIs and ICIs. In this study, we validated the prognostic value of CRAFITY score in HCC patients treated with the combination therapy, and found that CRAFITY score could be the OS- and PFS-related predictive factor, and may be associated with the tumor response and AEs. The CRAFITY score composed of AFP and CRP could stratify the PFS of HCC patients. To our knowledge, this is the first study assessing the utility of the CRAFITY score in HCC patients treated with the TACE plus TKIs and PD-1 inhibitor.

The CRAFITY score was an independent prognostic factor for OS and PFS in this study. Yang and colleagues evaluated the applicability of the CRAFITY score in a lenvatinib-immunotherapy combination cohort of 108 patients and a lenvatinib-treated. These two paragraphs should be connected together cohort of 72 patients with HCC, and the results showed that the CRAFITY score successfully predicted OS in the three stratifications [8]. Similarly, Hatanaka et al. evaluated the utility of CRAFITY score in 297 HCC patients treated with atezolizumab plus bevacizumab [11], and found that lower CRAFITY score was associated with the better PFS and OS, which was consistent with the results in this study.

Inflammation is considered a hallmark of cancer progression and a key component of the tumor microenvironment [12, 13]. AFP is a widely used tumor biomarker in the clinical practice and is considered as a positive predictor for the recurrence and the progression of HCC [3–5]. Currently, the role of AFP in immunotherapy has attracted much attention. Spahn et al. Including 67 HCC patients receiving nivolumab and 32 HCC patients receiving pembrolizumab, lower AFP levels were found to be associated with longer median PFS and OS [14]. Hsu et al. also identified that the AFP response was an independent predictor of PFS [15]. Likewise, a decrease in AFP levels post-treatment has been shown to be a predictor of prognosis [16, 17]. In summary, AFP exerts its immunosuppressive effect through two indispensable pathways: on the one hand, extracellular AFP induces the immune cells apoptosis and weaken their antitumor function [18]. On the other hand, extracellular and intracellular AFPs promote the upregulation of the immunosuppressive ligands or antigens, thereby promoting the immune escape [19].

Recent evidence indicates that CRP is closely related to tumor immunosuppression. CRP is an acute protein induced and regulated by interleukin 6, produced in the liver, and plays an important role in the innate and adaptive immune system [20, 21]. Zhang and coworkers demonstrated that the elevated levels of CRP could inhibit the Th1 differentiation and augmented Th2 differentiation of CD4 T cells [22]. To date, many studies reported that CRP is a novel prognostic marker in HCC patients. Hatanaka et al. revealed that the CRP was a predictive factor of PFS and OS in patients treated with atezolizumab and bevacizumab [11]. Zhang et al. found in 101 HCC patients receiving PD-1 inhibitor therapy that pre-treatment CRP levels have great potential for determining the effectiveness of ICIs [23]. There previous reports support the present findings that CRP is a predictive factor for poor tumor response and shorter PFS.

The changes in the local tumor immune microenvironment and systemic response after TACE are quite complex. However, numerous studies have shown that TACE has a negative impact on tumor immune response. Pinato et al. found that TACE is associated with a decrease in the density of immune-exhausted effector cytotoxic cells and T-regs within the tumor, along with a significant upregulation of pro-inflammatory pathways [24]. This highlights the diverse effects of TACE in modulating the tumor microenvironment. On the other hand, Tan et al. reported an increase in tumor-associated macrophages (TAMs) and a decrease in the number of CD8 + T cells in the post-TACE microenvironment [25]. TACE treatment involves embolization and chemotherapy. The purpose of embolization is to interrupt blood flow to the tumors, inducing ischemic necrosis and tumor hypoxia. However, due to the complexity of the blood supply in HCC and the limitations of interventional embolization technology, embolization of tumor-feeding blood vessels may be incomplete. This results in a reduction, rather than a complete blockage, of the tumor’s oxygen supply, leading to the formation of a hypoxic microenvironment. The presence of a hypoxic microenvironment inhibits macrophage function, weakens the ability of immune effector cells, and hampers the ability of dendritic cells to process tumor antigens and present them to lymphocytes [26]. On the other hand, hypoxia can also directly increase the expression of PD-L1 in myeloid-derived suppressor cells, dendritic cells, and cancer cells by activating HIF-1α. This participation in immunosuppression and immune evasion has been observed [27, 28]. However, the impact of chemotherapy on immune response is two-fold. Research has shown that chemotherapy not only leads to cell death in the immune system and negative effects, but also enhances the immune response by reducing immunosuppressive elements in the tumor microenvironment and improving the performance of antigen-presenting cells [29]. Similarly, higher CRAFITY scores also indicate an immune-suppressive intra-tumoral immune microenvironment characterized by a decrease in effector immune cells and an increase in suppressive immune cells. Based on these previous studies, it is reasonable to combine AFP and CRP in HCC patients undergoing TACE plus TKIs and ICIs. Our results also confirm the prognostic value of the CRAFITY score in this particular group of patients.

The other risk factor for OS is TACE sessions. There seems to be a question of what is cause and what is effect that needs to be resolved. We thought it might be more convincing that the more TACE a patient underwent, the longer the patient survived. Many studies have aimed to evaluate the efficacy of TACE in combination with other therapeutic approaches (such as ablation, TKI, and ICI) in the treatment of advanced HCC, better than any monotherapy [30–32]. The goal of TACE in HCC patients is to achieve local control or tumor shrinkage. Barynik et al. identified that the longer time to achieve local tumor control was associated with the poor treatment outcome [33]. In this study, we again demonstrate that TACE was a major part of the treatment of HCC and that TACE should be administered “on demand” to maximize clinical benefit.

Results regarding tumor radiological response remain controversial. Scheiner et al. reported that the CRAFITY score could predict the DCR and ORR in the immunotherapy patients, but not in sorafenib-treated patients [7]. Hatanaka et al. showed that there was a significant difference in DCR, but there was no significant difference in ORR [11]. However, Yang et al. found that no significance difference was observed in DCR and ORR in patients with different CRAFITY scores, but there was a downward trend in the combined treatment group and the lenvatinib treatment group [8]. In this study, we observed that at the 3-month evaluation of tumor response, the group with lower CRAFITY scores achieved a higher ORR compared to the groups with intermediate and high CRAFITY scores. However, there was no statistically significant difference in DCR among the groups. Conversely, at the 12-month evaluation, there was a statistically significant difference in DCR, but not in ORR. At the 6-month follow-up, neither ORR nor DCR showed any statistical significance. These findings suggest that the CRAFITY score may have limited predictive power in assessing tumor response.

The mechanisms underlying the association of CRAFITY score and AEs remains unclear. Hatanaka et al. reported the more frequency of decreased appetite, protein urea, fever, fatigue in all the patients and that grade ≥ 3 liver injury occurred more frequently in the CRAFITY 2 points group [11]. Similarly, we also found that the protein urea was more likely to appear in the CRAFITY-high group. CRP has been reported to be associated with immune-related AEs (irAEs) in patients receiving immunotherapy. Yu et al. showed that the elevated CRP level was associated with the onset of irAEs in patients with liver cancer receiving ICIs and TKIs therapy [34]. As far as we know, the relationship between serum AFP levels and the occurrence of AEs has not been studied. Therefore, the CRAFITY score may have a potential predictive ability for the occurrence of AEs in HCC patients, which deserves further validation.

Our study had several limitations. Firstly, this is a retrospective study, which is inevitably subject to the biases that affect this type of research. Secondly, it is worth noting that only anti-PD-1 inhibitors were utilized in this study. Therefore, it is recommended that future studies incorporate HCC patients who have received other ICIs to further validate the predictive capability of the CRAFITY score. Lastly, it should be mentioned that the statistical analysis did not include the degree of histologic differentiation due to the insufficient number of biopsy-proven HCCs in the study cohorts.

In conclusion, our study demonstrated the predictive value of the CRAFITY score in HCC patients treated with TACE plus TKIs and ICIs. The CRAFITY score is objective and simple in identifying the candidates for clinical trial inclusion and support decision-making in clinical practice.

## Data Availability

All data are available from the corresponding author upon request.

## Abbreviations

HCC: Hepatocellular carcinoma
TACE: Transarterial chemoembolization
OS: Overall survival
ICIs: Immune checkpoint inhibitors
TKIs: Tyrosine kinase inhibitors
AFP: Alpha-fetoprotein
CRP: C-reactive protein
CSM: CalliSpheres® microspheres
PD-1: Programmed death 1
HAIC: Hepatic arterial infusion chemotherapy
AEs: Adverse events
PFS: Progression-free survival
ECOG: Eastern Cooperative Oncology Group
ALT: Alanine aminotransferase
AST: Aspartate aminotransferase
PLR: Platelet/lymphocyte ratio
NLR: Neutrophil/lymphocyte ratio
BCLC: Barcelona Clinic Liver Cancer
mRECIST: Modified Response Evaluation Criteria in Solid Tumors
CR: Complete response
PR: Partial response
SD: Stable disease
PD: Progressive disease
ORR: Objective response rate
DCR: Disease control rate
iCPD: Immune-related confirmed progressed disease
iRECIST: Immune-related response criteria in solid tumors
irAEs: Immune-related adverse events
